# Spatiotemporal interaction characteristics and transition mechanism of tourism environmental efficiency in China

**DOI:** 10.1038/s41598-023-40047-2

**Published:** 2023-08-30

**Authors:** Zhenjie Liao, Lijuan Zhang

**Affiliations:** Guangzhou Huashang College, Guangzhou, 511300 China

**Keywords:** Ecology, Environmental social sciences

## Abstract

High-quality development is the theme of China’s economic and social development in the new era, and it is also an objective need for tourism development in the 14th Five-Year Plan period. This study presents an investigation of China’s patterns of tourism environmental efficiency from the perspective of spatiotemporal interactions. A nested analytical framework of quantile regression and spatiotemporal leaps was used to explore the driving mechanism patterns of tourism environmental efficiency under different leap types. Based on various spatial analysis methods, we posit that the patterns of tourism environmental efficiency differ through spatial associations, dynamic evolutions, and transition mechanisms. Our results indicate that there is a dynamic convergence trend of the overall differences in tourism environmental efficiency in China from 2000 to 2020 where a significant clustering phenomenon is observed in space and the level of spatial clustering gradually tends to be stable. In terms of local spatial structures and the dependence directions of tourism environmental efficiency, China’s northwest and northeast regions are more volatile, while eastern coastal regions are relatively stable. Spatiotemporal leaps of tourism environmental efficiency show certain transfer inertia with strong spatial dependence or path-locked characteristics, among which most central and western regions always maintain high carbon emission attributes. These regions are the most limited in the synergy of tourism environmental efficiency. The spatiotemporal network patterns of tourism environmental efficiency are mainly based on positive correlations and show strong spatial integration. However, a few neighboring provinces still have a certain degree of spatiotemporal competition. Driving patterns of the spatiotemporal leaps in tourism environmental efficiency among regions differ greatly. The eastern coastal provinces are driven by population-urbanization constraint patterns, and the northwest, southwest, and northeast regions are driven by technology regulation patterns. From the southeast to the northwest, the leap in the environmental efficiency of China's tourism gradually shows a stepwise pattern of "congruent constraint-reverse development-congruent development.” Therefore, the government should not only consider these various driving/constraining factors but also combine different environmentally-efficient tourism clustering types and transition paths to emphasize differentiated environmental tourism measures. This can help avoid the closure of inter-provincial tourism policies through inter-regional synergy.

## Introduction

Since the reform and opening up, China's economy gained a rapid growth of GDP and a series of achievements. But with the increase of the total amount and scale, the original power of economic growth will no longer meet the requirements of high-quality development in the new era. China's economic development has entered a new era, promoting regional integration and improving production efficiency are important support for realizing the transition from high-speed growth to high-quality development. From a broad perspective, the high-quality development of China's tourism industry requires improving the functional benefits of tourism and thus exerting positive external effects. From a narrow perspective, the high-quality development of China's tourism industry requires upgrading the quality of tourism products,improving the efficiency of tourism economy, optimizing the structure of tourism industry and enhancing the sustainability of tourism. Against the backdrop of achieving the "dual carbon goals", promoting green development of the tourism industry has become a dependent path for high-quality development of the tourism industry in the new era. As an important component of the national economic system, the tourism economy usually relies on large-scale investment in resources and energy. The inefficient and high consumption extensive tourism development model undoubtedly has caused many interferences to resources and the environment, leading to increasingly severe ecological and environmental problems. The contradiction between the development of tourism economy, resource allocation, and environmental protection is increasing day by day; the development of high-quality tourism places higher demands on the rational allocation of resources and the improvement of environmental efficiency. Therefore, there is an urgent practical need to study the efficiency of tourism environment.

Efficiency is the utility ratio between inputs and outputs of economic production factors that requires regional economic development to balance resource utilization and output effects^1^. Tourism efficiency refers to obtaining the maximum output with the minimal amount of tourism-related resource input within a certain time frame to satisfy stakeholders' needs for maximizing the total surplus. This can reflect the reasonableness of tourism growth^2^. The study of tourism efficiency is important for the high-quality development of tourism. The high growth of the tourism economy is valued by the academic community to coordinate its development; regional tourism differences and tourism spatial structure have become hot topics of research. In-depth research on the tourism industry, regional tourism development, ecotourism, and other related tourism efficiency topics^3^ has produced crucial results^4^. Most relevant studies consider regions with developed tourism such as Europe and the United States as case sites, select comparative factors to construct measurement models, determine regional tourism resource endowment^5^, tourism competitiveness^6^, and source markets^7^ based on static cross-sectional data or dynamic panel data, and describe their spatial differentiation characteristics^8^, causes^9^, and impacts^10–12^. Research perspectives include measuring tourism scale^13^, efficiency^14^, or performance^15^ from multi-level scale units, such as zones^2^, provinces^16^, municipalities^17^, and counties^18^, using single indicators such as tourism arrivals, revenue, and related increments or constructing comprehensive evaluation index systems and input–output indicators. Moreover, research methods used include scale distribution theory^19^, spatial field effect theory^20^, gravity model^21^, spatial econometric model^22^, as well as total factor productivity^23^, two-stage nested Thayer coefficient decomposition^24^, and exploratory spatial data analysis^25^ to explore the characteristics of spatiotemporal differentiation, spatial structure evolution, influencing factors, and driving mechanisms of tourism development^26–28^. Specifically, scholars' research on tourism efficiency focused on hotel management performance and operational efficiency. Kim et al.^29^ analyzed hotel efficiency, including customer satisfaction, and observed that the overall hotel efficiency value could better reflect hotel productivity. Arbelo-Pérez et al.^30^ used the stochastic frontier model to measure the efficiency of the hotel industry. Higuerey et al.^31^ used the network data envelope to assess the efficiency of the hotel industry in Ecuador. Subsequent related studies gradually extended to travel agencies and tourist attractions and destinations. Dragan et al.^32^ proposed an assessment model for travel agency efficiency. Abrate et al.^33^ used a two-stage data envelopment analysis to explore the operational efficiency of traditional travel agencies. Furthermore, Niavis^34^ evaluated the comprehensive spatio-temporal efficiency of tourist destinations in the Mediterranean coastal region. Based on a literature search, we found that a number of domestic and foreign scholars have studied tourism efficiency; however, relatively few studies have focused on the topic of tourism environmental efficiency. Environmental efficiency refers to the provision of products or services with competitive price advantages that meet people's pursuit of a happy life, while reducing the impact of products or services on the ecological environment and the intensity of resource consumption. This study considers tourism environmental efficiency as the ratio of input elements, such as tourism resources, labor, tourism capital, and environmental management, to output elements, such as tourism economic value and their impact on the environment in a complex tourism economic, resource, and environmental system. It measures the economic value generated by tourism economic activities and evaluates the impact caused by tourism economic development on the ecological environment. Higher tourism environmental efficiency value is correlated with greater tourism economic value and a lesser environmental load caused by tourism development.

Tourism efficiency has been studied systematically by scholars and it has a solid theoretical foundation in research. While many scholars have analyzed the spatial differentiation of tourism environmental efficiency based on geography, most studies have been limited to the cross-sectional characteristics of spatial differences and correlations and so far have not depicted spatial relationships, patterns, or changes from the perspective of spatiotemporal interactions. In terms of revealing the influencing factors, existing studies have mostly examined and analyzed the influencing factors of tourism environmental efficiency with the help of traditional decomposition methods or regression models. However, these tend to ignore the heterogeneity of driving factors at different levels of tourism environmental efficiency^35–38^.

This study investigates the spatiotemporal evolution and dynamic interaction characteristics of tourism environmental efficiency in China from 2000 to 2020 through the integrated application of multiple spatial analysis methods. Moran's *I* index, exploratory spatio-temporal data analysis, Quantile regression and other methods were used. We selected 2000–2020 as the study period. The indicators' data were obtained from the China Statistical Yearbook, China Environmental Statistical Yearbook, the China Tourism Statistical Yearbook, statistical yearbooks of provinces (autonomous regions and municipalities), and the official websites of provincial cultural and tourism departments between 2001 and 2021. A nested analytical framework of quantile regression and spatiotemporal leaps was used to explore the driving mechanism patterns of tourism environmental efficiency under different leap types. On the one hand, it is intended to improve the macroscopic cognition of the current situation of tourism environmental efficiency and its dynamic change patterns as well as reveal the driving modes of leap changes in tourism environmental efficiency under the influence of multiple factors. On the other hand, it will help to fully explore the potential of tourism environmental efficiency in practice, and provide a reference for the formulation of "common but differentiated" tourism environmental policies based on the differences in the effects of various driving modes under different leap types. Additionally, it may help to fully explore the potential of tourism environmental efficiency and provide a reference basis for the formulation of "common but differentiated" tourism environmental policies based on the differences in the effects of various driving models under different types of transition. This can serve the practical needs of constructing low-carbon tourism under a "double carbon" objective.

## Methods and data sources

### Moran's I index

There are many indicators and methods to measure spatial autocorrelation. Moran's I index is one of the most commonly used basic indicators, which can comprehensively characterize the average spatial correlation, spatial distribution pattern and significance of specific variables or attributes of each unit in the region. Therefore, the global Moran's *I* index in the spatial autocorrelation analysis method was used to analyze the spatial correlation and degree of agglomeration of tourism environmental efficiency in each province of China. The value range of Moran's *I* index is [-1, 1], and when the index value is 0, it indicates that there is no spatial correlation. When the value is closer to 1, it indicates a stronger positive spatial correlation between regional units and a higher degree of agglomeration. The specific formula is as follows:

1 $${\text{Moran's }}I = \frac{{n\sum\limits_{i = 1}^{n} {} \sum\limits_{j = 1}^{n} {w_{ij} \left( {x_{i} - \overline{x} } \right)\left( {x_{j} - \overline{x} } \right)} }}{{\sum\limits_{i = 1}^{n} {} \sum\limits_{j = 1}^{n} {w_{ij} \left( {x_{i} - \overline{x} } \right)^{2} } }},$$

where *n* is the number of study units (provinces), *x*_*i*_ and *x*_*j*_ are the tourism environmental efficiency values of neighboring provinces, $\overline{x}$ is the mean tourism environmental efficiency value, *w*_*ij*_ denotes the spatial weight matrix.

### Exploratory spatiotemporal data analysis (ESTDA)

Spatiotemporal interaction is an important method and perspective for geographers to study socio-economic development changes; however, previous studies on the spatial variation of geographic variables and their evolution have focused more on measuring cross-sectional characteristics^39^. The ESTDA method can effectively integrate time and space, which helps compensate for the lack of measurement in traditional spatial analysis methods in the time dimension and realizes spatiotemporal interaction analysis^40^.

#### LISA time path

The LISA time path reflects the geometric characteristics of the movement of geographic variables in LISA coordinates in the Moran scatter plot. The analysis of the pairwise migration of attribute values and spatially lagged values of tourism environmental efficiency in each province explains the characteristics of spatiotemporal synergistic changes and the dynamics of regional tourism environmental efficiency^41^. Specifically, two indicators, the relative length, and curvature were included^42^.

2 $$U = \frac{{N\sum\nolimits_{t = 1}^{T - 1} {} d\left( {L_{i,t} ,L_{i,t + 1} } \right)}}{{\sum\nolimits_{i = 1}^{N} {} \sum\nolimits_{t = 1}^{T - 1} {} d\left( {L_{i,t} ,L_{i,t + 1} } \right)}},$$

3 $$\beta = \frac{{\sum\nolimits_{t = 1}^{T - 1} {} d\left( {L_{i,t} ,L_{i,t + 1} } \right)}}{{d\left( {L_{i,1} ,L_{i,T} } \right)}},$$

where: *U* is the relative length; *β is the* curvature; *N is the* number of study units; *d*(Li, t, Li, t + 1) is the distance of study unit *i* moving during the period from *t* to t + 1. If *U* and* β* are greater than 1, it indicates that the study unit has a more dynamic local spatial structure and dependence direction during the study period, and vice versa.

#### Space–time leap

To further examine the transfer states and dynamic paths of local spatial association types of tourism environmental efficiency, this study draws on spatiotemporal leap theory^43^ and combines LISA temporal paths with traditional Markov chains to form a spatiotemporal leap matrix, which mainly includes four types: type I (only self-leap), type II (only domain leap), type III (both self and neighborhood leap), and type IV (both self and neighborhood remain stable) (Table 1). Based on the types of leaps in the efficiency of the tourism environment in each region, spatial cohesiveness can be calculated, that is, the proportion of the number of units in which a type IV leap occurs to all possible leap units.

**Table 1:** Space–time transition type.

Type	Space–time transition form	Symbolic representation
I	Self transition-neighborhood stability	HH_*t*_ → LH_*t*+1_, LH_*t*_ → HH_*t*+1_, HL_*t*_ → LL_*t*+1_, LLt → HL_*t*+1_
II	Self stability-neighborhood transition	HHt → HH_*t*+1_, LHt → LH_*t*+1_, HLt → HL_*t*+1_, LLt → LL_*t*+1_
III	Self transition-neighborhood transition	HHt → LH_*t*+1_, LHt → HH_*t*+1_, LLt → HL_*t*+1_, HLt → LL_*t*+1_
IV	Self stability-neighborhood stability	HHt → HH_*t*+1_, HHt → HH_*t*+1_, LLt → LL_*t*+1_, LLt → LL_*t*+1_

#### Spatiotemporal interaction visualization

The spatiotemporal interaction characteristics of socioeconomic development changes can also be characterized by introducing graph theory^44^ to compensate for the loss of certain insignificant correlation information in traditional spatial analysis. This study calculates the covariance coefficients of the LISA spatiotemporal movement trajectories between each province and neighboring provinces and visualizes them in the form of a spatiotemporal topological network to reveal the degree of similarity in the competing dynamics and development mechanisms between provinces during the dynamic evolution of tourism environmental efficiency^45^.

Based on the covariance coefficients of LISA spatiotemporal trajectories, the dynamic correlations among neighboring provinces can be classified into four types: strong positive correlations (0.5 ~ 1), weak positive correlations (0 ~ 0.5), strong negative correlations (− 1 ~ − 0.5) and weak negative correlations (− 0.5 ~ 0). When the value of the covariance coefficient is greater than 0, it is a positive association state, indicating that neighboring provinces are in a positive synergistic development state in the process of spatiotemporal change; when the value is less than 0, it is a negative association state, indicating that there is a certain degree of spatiotemporal competition between neighboring provinces; and when the value is 0, there is no dynamic association relationship between neighboring provinces.

### Quantile regression

Unlike traditional OLS estimation, quantile regression is used to estimate the parameters by minimizing the sum of the absolute values of the weighted residuals. Thus, it is less susceptible to the constraints of the distribution assumptions and outliers^46^, and the estimation is more robust. At the same time, the model portrays the conditional distribution more carefully and can be deeply nested with different types of spatiotemporal leaps^47^, which helps resolve and discriminate the driving mechanisms of spatiotemporal leaps in tourism environmental efficiency at different quantile levels. The details are as follows:

Assume that the probability distribution of variable* Y* is:

4 $$F\left( y \right) = \Pr ob\left( {Y - y} \right).$$

The quantile of *τ* is defined as:

5 $$q(\tau ) = \inf \left\{ {y:F(y) \ge } \right\},0 < \tau < 1$$

Then the minimum objective function of quantile *q(τ)* of *F(y)* is:

6 $$\begin{aligned} q(\tau ) = & \arg \min_{\zeta } \left\{ {\tau \int\limits_{y > \zeta } {\left| {y - \zeta } \right|dF(y) + (1 - \tau )\int\limits_{y < \zeta } {\left| {y - \zeta } \right|dF(y)} } } \right\} \\ = & \arg \min_{\zeta } \left\{ {\int {_{\rho \tau } } \left( {y - \zeta } \right)dF(y)} \right\}. \\ \end{aligned}$$

### Selection of influencing factors and data sources

#### Selection of influencing factors

Tourism environmental efficiency is a comprehensive reflection of the coordinated development of tourism and environment systems. Tourism environmental efficiency should comprehensively measure the input and output elements of tourism and the environment. Based on the principles of importance, comparability, scientificity, accessibility of the selected indicators, and previous studies, the input–output evaluation index system of tourism environmental efficiency was constructed (Table 2). Among them, the input indicators were selected from the tourism enterprises (including A-class tourist attractions, travel agencies, and star-rated hotels), the number of tourism employees, investment in tourism fixed assets, and investment in environmental pollution control. The output indicators were divided into desired and non-desired outputs. The desired output was from the total tourism revenue, while the non-desired output was the tourism carbon emission. Due to China's tourism statistics system not being sound, the current statistical yearbook data only involves the data of tourism enterprises such as A-class tourist attractions, travel agencies, and star-rated hotels. Therefore, we selected A-class tourist attractions, travel agencies, star-rated hotels, and other tourism, the number of enterprises scales, and tourism fixed asset investment as input indicators. The total investment in environmental pollution control was the input index of environmental control. Tourism carbon emissions were measured using the "bottom-up" method, which sums up the carbon emissions from tourism transportation, accommodation, and activities. Drawing on the empirical research methods of Becken et al.^48^ and Patterson et al.^49^, tourism transportation, tourism accommodation, and tourism activities are identified as the key areas of CO_2_ emissions in the tourism industry. The decomposition and aggregation method is adopted to measure the CO_2_ emissions in the tourism industry from the bottom up. The specific calculation method is as follows:

7 $$C^{t} = \sum\limits_{j = 1}^{3} {C_{j}^{t} } = C_{1}^{t} + C_{2}^{t} + C_{3}^{t} .$$

**Table 2:** Evaluation indicator system of tourism environmental efficiency.

Type	Indicator name	Indicator description
Input variables	Resource input	Scale of tourism enterprises (total number of A-level tourist attractions, travel agencies and star hotels)
	Capital investment	Investment in tourism fixed assets (total investment in fixed assets of A-level tourist attractions, travel agencies and star hotels)
		Total investment in environmental pollution control
	Labor input	Number of tourism employees
Output variables	Expected output	Total tourism income
	Unexpected output	Tourism carbon emissions (the sum of carbon emissions from tourism transportation, tourism accommodation and tourism activities)

In the formula, *C*^*t*^ represents the total CO_2_ emissions (g) from the tourism industry in year *t*; $C_{j}^{t}$ represents the CO_2_ emissions (g) of sector *j* in year *t*; $C_{1}^{t}$ represents the CO_2_ emissions from tourism transportation in year *t* (g); $C_{2}^{t}$ represents the CO_2_ emissions (g) from tourism accommodation in year *t*; $C_{3}^{t}$ represents the CO_2_ emissions (g) from tourism activities in year *t*.

Geodetector is a new statistical method to detect spatial heterogeneity and reveal the driving mechanism behind it and is now widely used in studies related to the interaction mechanism between socio-economic factors and natural environmental factors. In this study, the geodetector model was used to explore the factors influencing tourism environmental efficiency in China, investigate the driving mechanism of the factors on tourism environmental efficiency, and further analyze the interaction of different influencing factors. The geodetector approach was used to identify the main factors influencing tourism environmental efficiency and to analyze the two-by-two interaction of each influencing factor. There are numerous factors affecting tourism environmental efficiency^50^, and the level of economic development (*E*), industrial support capacity (*I*), urbanization level (*U*), technology development (*T*), transportation accessibility (*TA*), openness degree (*O*), environmental regulation level (*ER*), environmental self-cleaning capacity (*ES*), the relative abundance of tourism resources (*TR*), and human capital (*H*) are selected as the ten indicators used as detection factors (Table 3). Each detection factor was spatially adapted to the spatial distribution of tourism environmental efficiency levels in that year, to detect the influence of each factor in the study period.

**Table 3:** Influencing factors and indicators of tourism environmental efficiency in China.

Code	Detection factor	Specific indicators
*E*	economic development	GDP
*I*	industrial support	Proportion of total tourism revenue in the tertiary industry/%
*U*	urbanization level	Population urbanization rate/%
*T*	technology development	Total R&D investment
*TA*	transportation accessibility	Passenger turnover (/100 million people·km)
*O*	openness degree	Proportion of total import and export trade in GDP/%
*ER*	environmental regulation	Proportion of total investment in environmental pollution control to GDP/%
*ES*	environmental self-cleaning	Forest coverage/%
*TR*	tourism resources	The ratio of the absolute abundance of tourism resources to the area of each region
*H*	human capital	College students/10,000

#### Data sources

We selected 2000–2020 as the study period. The indicators' data were obtained from the China Statistical Yearbook, China Environmental Statistical Yearbook, the China Tourism Statistical Yearbook, statistical yearbooks of provinces (autonomous regions and municipalities), and the official websites of provincial cultural and tourism departments between 2001 and 2021. Missing data were obtained using the Linear interpolation method. The basic geographic data were mainly from the 1:4 million databases of the National Geographic Information Center. Individual missing data were supplemented by interpolation, and the relevant economic data were uniformly converted to constant 2000 prices. In addition, the variables were normalized before the empirical analysis to reduce heteroscedasticity and nonstationarity.

## Results

### Time-series evolution and spatial correlation analysis of tourism environmental efficiency in China

**Figure 1:**
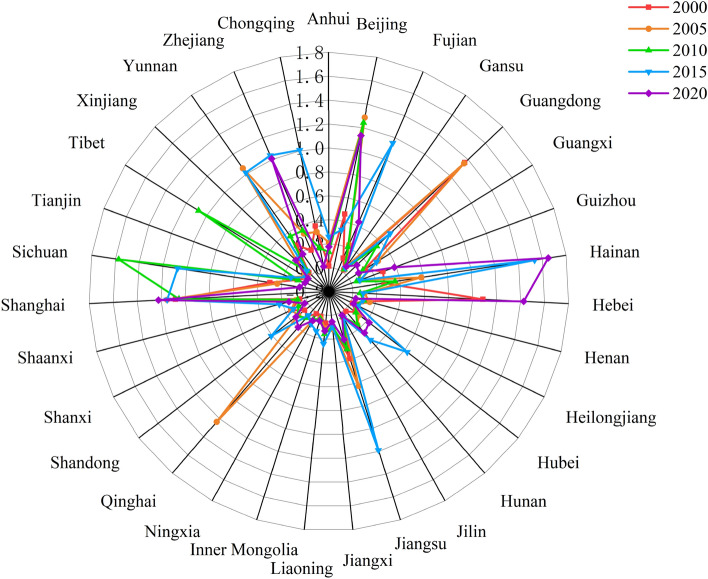
Changes in tourism environmental efficiency of China.

Based on DEASOLVER Pro 5.0 software, the non-radial (non-oriented), variable scale payoff (VRS) super-efficient SBM model was used to measure the tourism environmental efficiency of 31 provinces and cities in China in 2000–2020, estimate the average yearly value to conduct a comparative analysis of different regional tourism environmental efficiencies. The mean efficiency values were compared and analyzed (Fig. 1). Overall, the number of regions with tourism environmental efficiency higher than the national average decreased from 2000 to 2020, with the most pronounced decrease in most of the eastern provinces and some regions in the south-central region. Nevertheless, the overall tourism environmental efficiency in China is still uneven, with regional differences and polarization.

**Figure 2:**
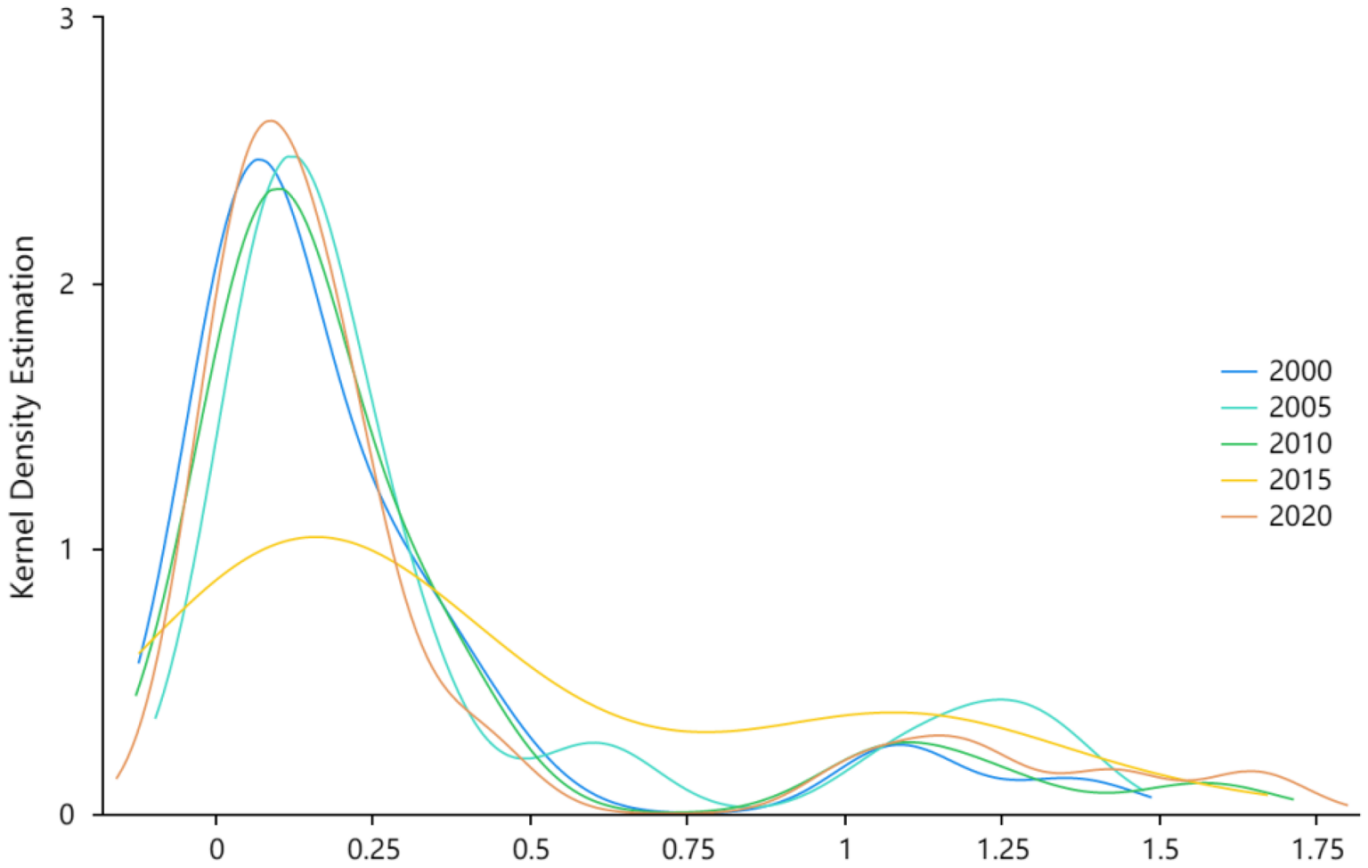
The Kernel density estimation of tourism environmental efficiency in China.

To further understand the overall difference in tourism environmental efficiency in China and its dynamic evolutionary characteristics, the kernel density estimation method was applied to analyze tourism environmental efficiency in 2000, 2005, 2010, 2015, and 2020, and the kernel density curve was plotted (Fig. 2). From the distribution position, the main peak of the kernel density curve from 2000 to 2020 shows a trend of shifting right and left, indicating that the overall level of China's tourism environmental efficiency increased and then declined during the sample period, which confirms the objective facts in the previous section. In terms of the distribution pattern, the peak of the main peak decreases and then rises during the study period, and the crest pattern also shows the evolution characteristics of "steep-flat-steep,” which indicates that there is a dynamic convergence trend of the overall difference of tourism environmental efficiency in China. The distribution extension shows a trailing phenomenon to the right, and the extension has a widening feature, indicating that the spatial gap between the high-value areas of China's tourism environmental efficiency is gradually expanding. In general, the dispersion degree of China's tourism environmental efficiency increases and then decreases; however, at the same time, the polarization effect appears in a few high-carbon emission regions, and the gap with other regions becomes increasingly larger.

The kernel density curve reflects only the regional differences and time-series variation characteristics of tourism environmental efficiency in China and further explores the spatial correlation characteristics of tourism environmental efficiency using the global Moran's *I index. As* can be seen from Table 4, Moran's *I index* is positive during the period 2000–2020, indicating that the tourism environmental efficiency of each province has a positive spatial correlation, and the spatial clustering phenomenon is more obvious. From the change of the study period, the global Moran's *I* index shows three stages of "decline-rise-stability,” which indicates that the spatial correlation of tourism environmental efficiency has weakened in the early stage of the study, but gradually stabilized in the later stage, and the spatial clustering The level of spatial concentration is becoming less and less volatile.

**Table 4:** Moran's I for tourism environmental efficiency in China.

Year	Moran's I	Z-score	Year	Moran's I	Z-score
2000	0.02203	0.105228	2011	0.097858	1.539401
2001	0.027131	0.742646	2012	0.029076	0.843137
2002	0.042516	0.942306	2013	0.229479	2.966065**
2003	0.103158	1.551116	2014	0.22011	2.875165**
2004	0.093501	1.493332	2015	0.175642	2.367464*
2005	0.100345	0.778169*	2016	0.177149	2.501608*
2006	0.088405	1.409908	2017	0.153606	2.208218*
2007	0.056438	1.029863*	2018	0.050973	1.064929
2008	0.103605	0.936935*	2019	0.052039	0.997802
2009	0.156406	2.169472**	2020	0.055598	1.057043
2010	0.166376	1.626923			

* indicates p<0.1, * * indicates p<0.05, and * * * indicates p<0.01.

### Spatiotemporal interaction characteristics of tourism environmental efficiency in China

#### LISA time path analysis

The relative lengths and curvatures of the LISA temporal paths were calculated using Eqs. (1) and (2), which can further verify whether Chinese tourism environmental efficiency has a stable local spatial structure and a spatially dependent direction^51^.

**Figure 3:**
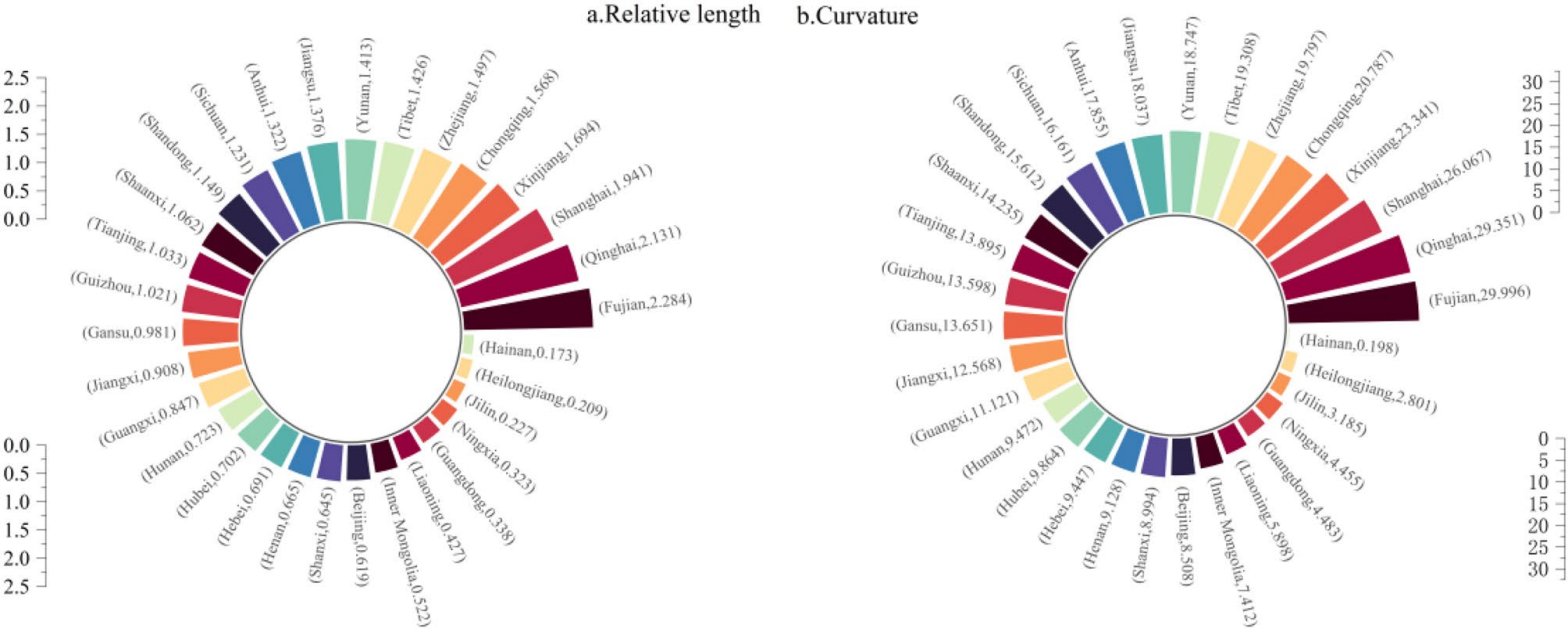
Geometric characteristics of LISA time path.

As shown in Fig. 3a, the relative lengths of Zhejiang, Chongqing, Xinjiang, Shanghai, Qinghai, and Fujian are all greater than the national average, indicating that their local spatial structures are highly dynamic. This is mainly because these regions are in a critical period of industrial restructuring, and while vigorously developing their tourism economies, their tourism environmental efficiency shows strong local spatial fluctuations. The relative lengths in Hainan, Heilongjiang, Jilin, Ningxia, Guangdong, Liaoning, and Inner Mongolia were shorter, mostly < 0.6. These provinces and regions are mainly in the developed eastern coastal areas, where tourism accounts for a higher proportion and has formed a more stable and intensive development mode, thus constituting a relatively stable local spatial structure. In general, the relative length of the LISA in most regions of the country is small, and overall, it shows a more stable local spatial pattern.

As shown in Fig. 3b, the curvatures of the central and western regions, such as Sichuan, Yunnan, Tibet, Chongqing, Xinjiang, and Qinghai, are all above 2, far exceeding the national average and showing a more volatile spatially dependent direction. This may be because most of these regions are close to the agglomeration centers or critical areas of high- and low-carbon emission areas; thus, the dynamic change process of tourism environmental efficiency is more spatially influenced by the domain, showing a dynamic direction of change and spatial dependence. The eastern coastal regions, such as Beijing, Liaoning, Guangdong, Jilin, Heilongjiang, and Hainan, have the smallest curvature, mainly because of the high similarity of tourism development patterns and energy consumption structures between these regions and neighboring domain provinces, and the fluctuations of spatial dependence are mutually held and influenced; thus, the curvature is smaller.

#### LISA spatiotemporal transition analysis

The spatiotemporal leap matrix can be used to further analyze the mutual transfer status of local spatial association types. As shown in Table 5, the spatial association pattern of tourism environmental efficiency in China from 2000 to 2020 was relatively stable, with fewer leaps between different types, showing a certain overall transfer inertia. Among the four types of spatiotemporal transition, the number of provinces belonging to type III was the largest and the spatial cohesion was high, indicating that the local spatial structure of tourism environmental efficiency in each province had obvious path-dependent characteristics throughout the study period. Among them, Hainan, Shaanxi and Shanxi all belong to the types HH_*t*_ → HH_*t *+ 1_ and HL_*t*_ → HL_*t* + 1_, indicating that the high carbon emission attributes are very stable in most of the central and western regions, which are the key regions limiting the synergy of tourism environmental efficiency. Overall, the tourism environmental efficiency of most Chinese provinces is less affected by the spatial influence of the domain, and its factors have a stronger influence on the spatial correlation structure, with strong path-dependent or spatially locked features.

**Table 5:** Spatiotemporal transition matrices of tourism environmental efficiency in China.

Transition type	HH_*t*+1_	HL_*t*+1_	LH_*t*+1_	LL_*t*+1_
HH_*t*_	IV (Hainan)	II (Sichuan,Chongqing)	I (Inner Mongolia,Heilongjiang,Jilin,Liaoning)	III (Jiangxi,Henan, Shandong,Guangdong)
HL_*t*_	II (Hubei,Gansu)	IV (Shaanxi,Shanxi)	III (Xinjiang,Qinghai,Tibet,Yunnan, Ningxia)	I
LH_*t*_	I	III	IV	II
LL_*t*_	III (Hunan,Guizhou)	I (Shanghai,Jiangsu, Zhejiang,Anhui,Fujian)	II	IV (Beijing,Tianjin, Hebei)

#### Spatiotemporal interaction visualization

**Figure 4:**
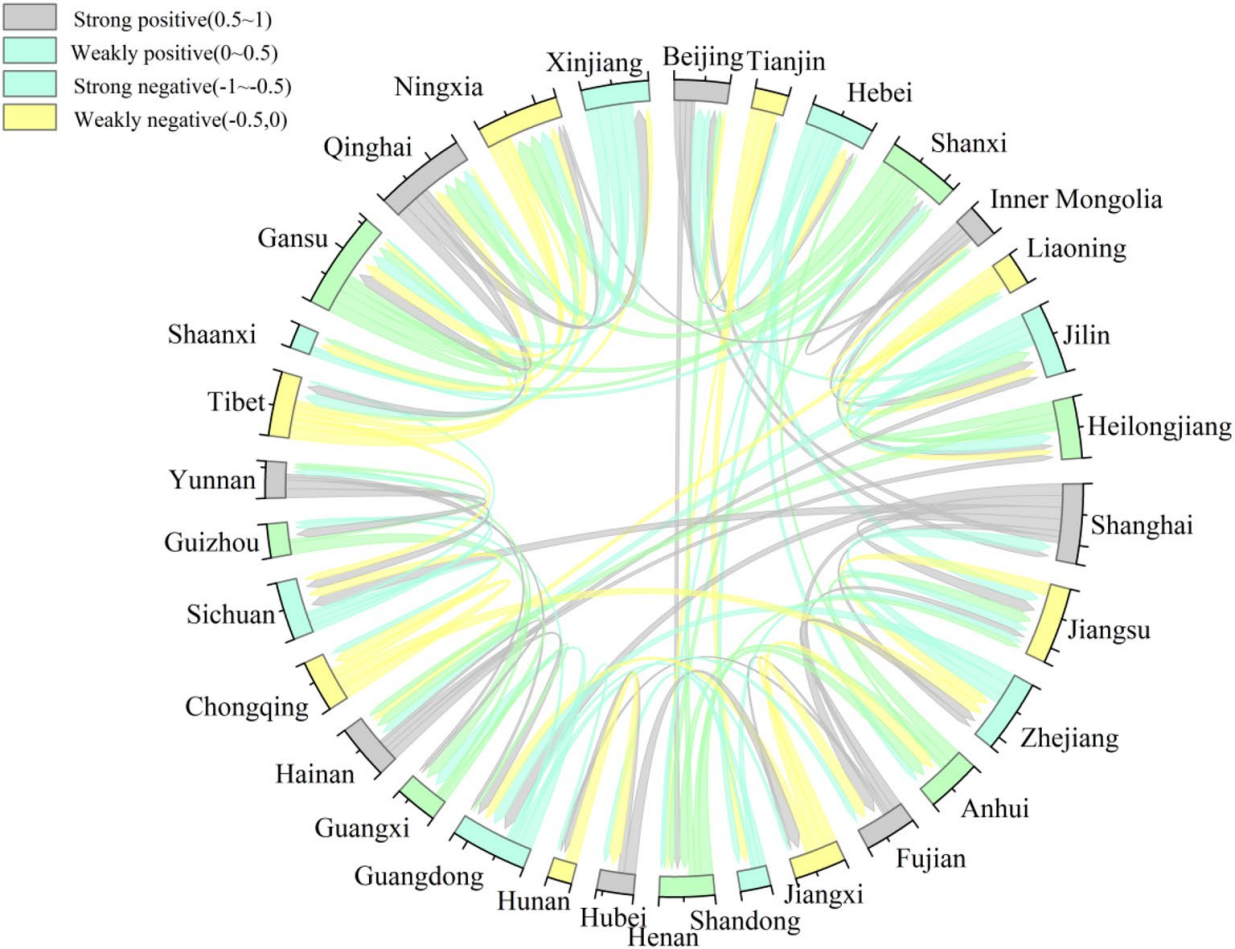
Spatiotemporal topology network of tourism environmental efficiency in China.

In the process of economic development and tourism, there may be spatiotemporal competition or a cooperation-win–win relationship between neighboring provinces. This study further explored the spatial association characteristics between neighboring provinces using a spatiotemporal topological network diagram. The spatiotemporal topological relationship is the foundation for defining new spatial objects, providing a feasible explanation for describing the relationships between new spatial objects and other spatial objects. It also involves the transformation and deformation of objects, as well as the connections between objects. It plays a crucial role in Geographic Information Systems (GIS) as it can define the position and properties of spatial objects in spatial structures. As shown in Fig. 4, the spatiotemporal network pattern of tourism environmental efficiency is mainly dominated by positive associations, and there are only 11 pairs of negative associations, accounting for less than 20%, indicating strong spatial integration in the process of spatiotemporal variation in tourism environmental efficiency in China. Among all the negative associations, Tianjin and Hebei, Yunnan and Guizhou, Qinghai and Tibet, Jilin and Heilongjiang, Gansu and Shaanxi had strong negative associations, reflecting the uneven characteristics of tourism environmental efficiency among neighboring provinces. These regions have a certain degree of spatiotemporal competition in the evolution of tourism environmental efficiency, which may be due to the closed nature of regional tourism environmental policies or the long-standing spatial incoherence between neighboring provinces, such as the existence of spatial heterogeneity in regional economic development levels, natural resource endowments, and the imbalance of supporting factors, such as low-carbon construction investment and low-carbon technologies, which eventually leads to further intensified inter-provincial competition. On the other hand, Beijing and Tianjin, Zhejiang and Fujian, Zhejiang and Guangdong, Sichuan and Chongqing show strong positive correlations. The evolutionary process of tourism environmental efficiency in these neighboring provinces has a high degree of similarity, and a geographically concentrated dynamic emerges, thus becoming a positive synergistic development region. In general, the development of low-carbon tourism actions needs to be integrated from a regional perspective, avoiding the closure of inter-provincial tourism environmental policies, and promoting overall regional collaboration.

### Analysis of the efficiency leap mechanisms of China's tourism environment

#### Quantile regression results

The level of economic development (*E*), industrial support capacity (*I*), urbanization level (*U*), science and technology development level (*T*), transportation accessibility (*TA*), openness to the outside world (*O*), environmental regulation level (*ER*), environmental self-cleaning capacity (*ES*), tourism resource abundance (*TR*), and human capital (*H*) were included in the quantile regression model, and drawing on related studies^52^, a total of five quartiles of 0.1, 0.25, 0.5, 0.75, and 0.9 were selected for estimation. To reduce pseudo-regression, a smoothness test was required before the empirical analysis. In this paper, the LLC and Fisher-ADF unit root tests were selected to test the smoothness of each variable (Table 6), and all variables were found to pass the significance test at the 1% level, indicating that the variables are smooth, indicating that the variables are smooth and can be directly estimated as parameters. In addition, the mean–variance inflation factor (VIF) of each variable indicator was 4.04, with a maximum value of 8.65, which is below the alert value of 10, so the problem of multicollinearity can be ignored in the regression analysis. From the estimation results of the quantile regression (Table 7), the industrial support capacity shows different degrees of negative effects on tourism environmental efficiency at each quantile; the level of economic development shows a positive promoting effect at the high quantile stage, but its quadratic coefficient is negative, indicating that there is an inflection point for the influence of the level of economic development on tourism environmental efficiency; the level of scientific and technological development shows a positive driving effect, and it increases with the quantile The level of scientific and technological development shows a positive driving effect, and increases with the increase of the quantile; the environmental regulation does not pass the significance test at each quantile; the abundance of tourism resources promotes the increase of tourism environmental efficiency at the low quantile stage; the urbanization level always maintains the inhibitory effect at each quantile.

**Table 6:** Unit root test of variables.

Variable	*E*	*I*	*U*	*T*	*TA*	*O*	*ER*	*ES*	*TR*	*H*
LLC	249.325***	− 259.73***	92.002***	104.03***	149.04***	20.315***	140.39***	− 236.03***	174.092***	− 23.98***
ADF	173.071***	− 51.646***	− 171.297***	160.808***	231.87***	− 77.896***	267.916***	− 180.444***	19.003***	88.004***

* indicates p<0.1, * * indicates p<0.05, and * * * indicates p<0.01.

**Table 7:** Quantile regression and OLS estimates.

Variable	Quantile					OLS
	0.1	0.25	0.5	0.75	0.9	
ln*E*	0.003	− 9.435	0.167	0.002	0.003	0.002
ln*I*	− 0.07	0.001***	0.001*	0.093	− 7.653	− 16.133
ln*U*	− 1.696	− 0.381	0.092***	0.07***	− 4.324	252.816
ln*T*	0	− 2.297	0.064	− 0.001	− 0.001	− 0.001
ln*TA*	0	0	0	0	0	0
ln*O*	− 1.541	0	0	0.205	− 5.648	− 8.68
ln*ER*	0.001**	− 2.25	0.086**	0.001	0	0
ln*ES*	− 1.144	0	0	0.035	− 3.028**	− 1597.01
ln*TR*	2.942	− 1.572	0.032	1.644	5.184	5.018
ln*H*	− 0.001	2.714**	1.369***	0.006	0.022	0.029*

* indicates p<0.1, * * indicates p<0.05, and * * * indicates p<0.01.

#### Temporal leap and quantile regression nesting

According to the size of the quantile, the quantile regression can be divided into two stages, high quantile (0.5 ~ 0.9) and low quantile (0.1 ~ 0.5), while four quantile response types, high quantile constraint, high quantile drive, low quantile constraint, and low quantile drive, can be further constructed according to the direction of differential effects of influencing factors, which can then be deeply nested with the spatiotemporal leap types^53^ (Table 8). The characteristics of the high quantile constraint mainly inhibit the growth of the tourism environment high-efficiency zone, where the isotropic constraint indicates that neighboring provinces are also constrained, and reverse development indicates that neighboring provinces leap or remain in the high-value zone. The high quantile drive maintains tourism environment efficiency in the high-value area, where isotropic development indicates that it drives the tourism environment efficiency growth of neighboring provinces simultaneously, and reverse development indicates that it is not affected by an obvious drive effect. The characteristic of the low-quantile constraint is to maintain its own tourism environmental efficiency in the low-value area, where the same direction constraint indicates that the neighboring provinces are also constrained, and reverse development indicates that the neighboring provinces complete the upward leap or remain in the high-value area. The low quantile drive promotes the tourism environmental efficiency intensity zone to leap upward, where the same direction of development indicates that the neighboring provinces also leap or remain in the high-value zone, and the reverse development indicates that the driving effect on the neighboring provinces has not been fully played.

**Table 8:** The nesting of spatiotemporal transition and response type of quantile regression.

Quantile response type	Corresponding transition direction	Corresponding transition type
High quantile restriction	Homonymous constraint	HH_*t*_ → LL_*t*+1_, HL_*t*_ → LL_*t*+1_
	Reverse development	HH_*t*_ → LH_*t*+1_, HL_*t*_ → LH_*t*+1_
High bit drive	Development in the same direction	HH_*t*_ → HH_*t*+1_, HL_*t*_ → HH_*t*+1_
	Reverse development	HH_*t*_ → HL_*t*+1_, HL_*t*_ → HL_*t*+1_
Low quantile restriction	Homonymous constraint	LL_*t*_ → LL_*t*+1_, LH_*t*_ → LL_*t*+1_
	Reverse development	LL_*t*_ → LH_*t*+1_, LH_*t*_ → LH_*t*+1_
Low bit drive	Development in the same direction	LL_*t*_ → HH_*t*+1_, LH_*t*_ → HH_*t*+1_
	Reverse development	LL_*t*_ → HL_*t*+1_, LH_*t*_ → HL_*t*+1_

#### Driving mechanism model for space–time leap

**Figure 5:**
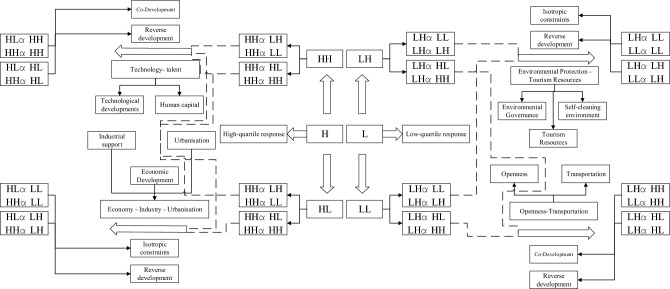
The driving mechanism of spatiotemporal transition of tourism environmental efficiency.

On the basis of the above analysis, the influencing factors of the high and low quantiles and the type of space–time transition are deeply nested, and four driving/restricting modes of space–time transition of tourism environmental efficiency are established (Fig. 5), which are open-traffic driven, environment-resource constrained, technology-human driven, economy-industry-urbanization constrained.

##### The environment-resource constraint model

Environmental protection and tourism resource abundance are the main factors that inhibit the growth of provinces with low tourism environmental efficiency, and the type of low-quantile constraints belong to this type of model. The environmental protection generally has a two-way impact on the tourism environmental efficiency. On one hand, environmental self-cleaning capacity can reduce the marginal abatement cost through the scale effect; on the other hand, an elevated level of environmental regulation may lead to diseconomies of agglomeration, and overcrowding causing all kinds of competitive costs to rise, thus generating excessive energy demand. In addition, environmental regulation also shows a positive driving effect on tourism environmental efficiency but fails to pass the significance test. The reasons may be that, first, environmental regulation, as a constraining force, increases the cost of pollution control for tourism enterprises, which may further crowd out their R&D investments and hinder energy efficiency and technological innovation; second, energy consumption in tourism in high quantile regions has increased significantly during the study period and still shows growth, resulting in the environmental regulation effect not yet fully manifested; third, it may be due to the government; third, it may be due to the lack of effective government regulation and performance control in the process of environmental governance, resulting in relevant regulatory instruments that have not yet formed effective constraints. Combined with the previous analysis (Tables 5 and 8), in the low-quantile stage, Beijing, Tianjin and Hebei were more affected by this constraint model, and tourism environmental efficiency always remained in the low-value area. Among them, most of the provinces on the eastern coast are of the same direction constraint type, and the tourism environmental efficiency of their domain provinces is similarly inhibited.

##### The openness transportation-driven model

The degree of openness to the outside world and accessibility are the main factors driving the leap to low-value agglomerations in tourism environmental efficiency; the low-quantile drive belongs to this model. In addition, the degree of openness to the outside world is significant in promoting the efficiency of the tourism environment. Accelerating the opening-up process and transportation accessibility can promote the agglomeration of talent and technologies in the service industry, and accelerate knowledge spillover and innovation resource sharing among service enterprises, all of which can help enhance tourism environmental efficiency. During the study period, China's tourism industry as a whole was in the scale expansion stage, and the industrial structure was still dominated by traditional high-energy-consuming low-end tourism, while the internal structure optimization and adjustment of the tourism industry were slow, and the "high technology" and "high knowledge-intensity" of the modern tourism industry The advantages of "high technology" and "high knowledge intensity" of the modern tourism industry have not yet played a significant role in constraining the efficiency of the tourism environment. Driven by the effect of this model, the original low-value area of tourism environmental efficiency gradually begins to leap upward, and only Hunan and Guizhou belong to this type, which is much smaller than the number of provinces in the low-quantile constraint type, indicating that the environmental resource constraint model has a broader influence in the low-quantile stage.

##### The economic-industrial-urbanization constraint model

High-quantile constraints are applied to the model. In this model, the industry support capacity and urbanization level still maintained a significant inhibitory effect on provinces with highly tourism environmental efficiency, and the constraint effect gradually increased as the quantile point increased. At the same time, the level of tourism economic development and its quadratic term both pass the significance test at the high quantile stage and show a "positive–negative" effect, indicating a non-linear relationship between tourism value added per capita and tourism environmental efficiency, which verifies to a certain extent that the development of service economy supports The "environmental Kuznets curve" hypothesis. Before the economic development level reaches the inflection point, tourism environmental efficiency increases with an increase in tourism value-added per capita. At this stage, the tourism industry shows a rough-scale expansion. When the added value of tourism per capita reaches a certain level, the growth mode of the tourism industry starts to become intensive, positive externalities such as economies of scale and knowledge spillover effects continue to emerge, emissions reduction efficiency increases and tourism environmental efficiency is further constrained. In addition, the level of urbanization had the same negative inhibitory effect on tourism environmental efficiency, passing the significance test at the 1% level. With the acceleration of the urbanization process and the promotion of intensive production and lifestyles, energy constraints and environmental pollution problems have become increasingly prominent, further inhibiting the improvement of tourism environmental efficiency. Combined with the previous analysis, Jiangxi, Henan, Shandong, Guangdong, Guangxi, Inner Mongolia, Heilongjiang, Jilin, Liaoning, Xinjiang, Qinghai, Tibet, Yunnan, Ningxia are significantly constrained by the effect of this model and gradually leap from the original high-tourism environmental efficiency area to the low-value area. Among them, Jiangxi, Henan, Shandong, Guangdong, Guangxi belong to the same direction constraint type, and the growth of tourism environmental efficiency in neighboring provinces is also hindered. Inner Mongolia, Heilongjiang, Jilin, Liaoning, Xinjiang, Qinghai, Tibet, Yunnan, Ningxia belong to the reverse development area, and the tourism environmental efficiency of neighboring provinces continues to grow without being affected by the inhibiting effect.

##### Technology-human powered

A high-quantile drive is applied to this model. Technological development has both positive and negative effects on energy consumption. From the empirical results of this paper, the level of technological development has a positive driving effect on tourism environmental efficiency. This may be because, on the one hand, changes in the level of technological development stimulate the economic activities of the tourism industry, which may induce new energy consumption and demand, making it difficult to form economies of scale in energy consumption. On the other hand, there may be time lags in the application and diffusion process of the level of technological development, and its emission reduction effect has not yet fully emerged. Among them, most of the central and western provinces are more influenced by this driving model, especially the western region, which shows an obvious homogeneous development trend. Environmental efficiency increases at the same time, driving the growth of tourism environmental efficiency in neighboring provinces, Hainan, Hubei, Gansu, and other places belonging to this type. While Sichuan, Chongqing, Shaanxi, Shanxi, and other places belong to the reverse development area, the neighboring provinces’ tourism environmental efficiency declines, and an emission reduction gap gradually emerges.

**Figure 6:**
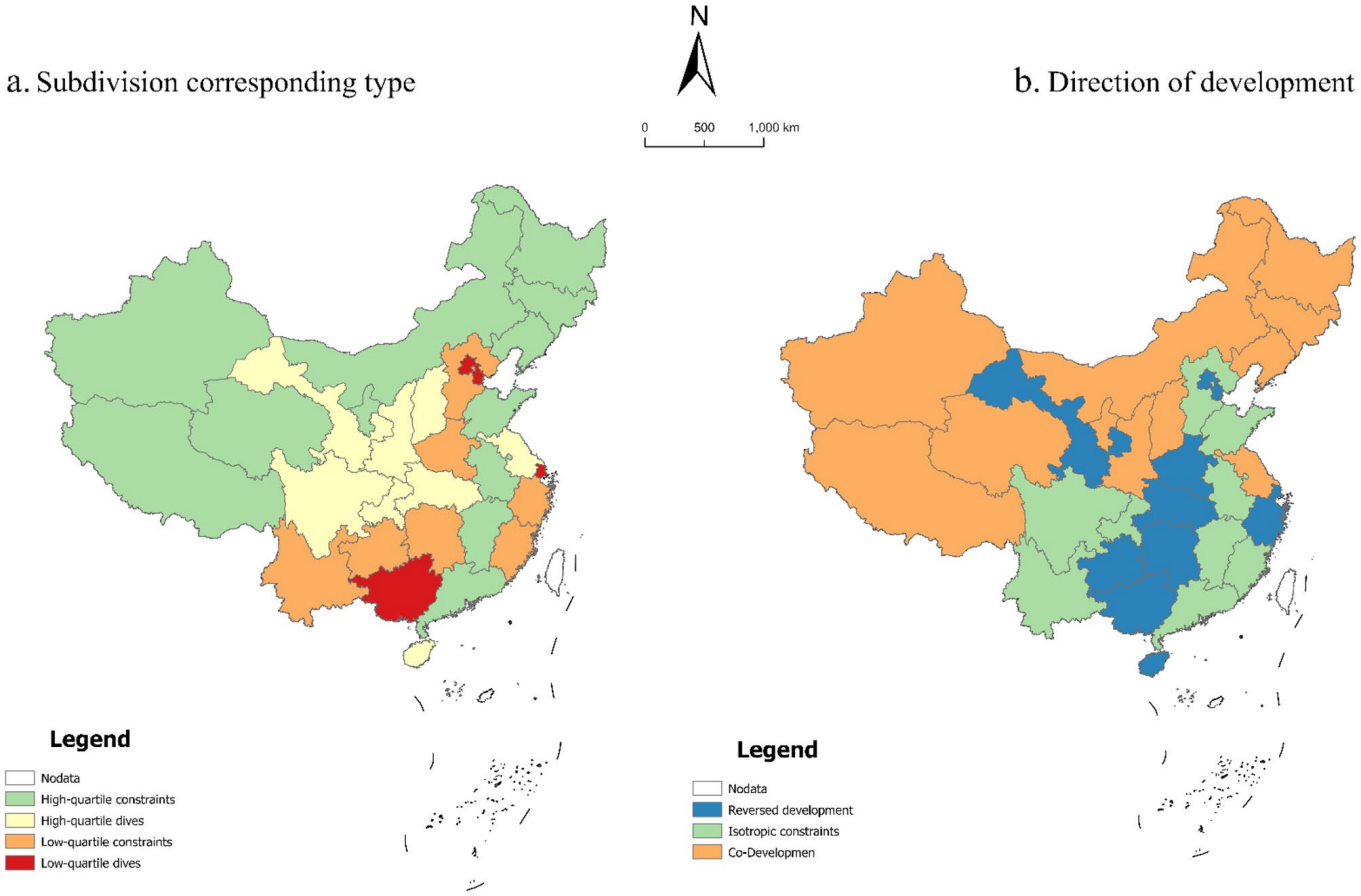
Spatial pattern of response types of quantile regression and development direction.

In general, from the southeast to the northwest, the leap pattern of China's tourism environmental efficiency gradually shows a stepwise progression of "congruent constraint—reverse development-congruent development" (Fig. 6). Among them, the eastern and southeastern regions have the same development trend and highly tourism environmental efficiency. Most of the provinces in the northwest and northeast mainly show the same direction constraint region, and the tourism environmental efficiency of the neighboring provinces in this region is maintained in the low-value area. From the quantile response type, the low quantile constraint is mainly distributed in the eastern coastal region, and the high quantile drive is mainly distributed in the northwest, southwest, and northeast, and these two drive/constraint types affect a wide range of provinces; only a few provinces are affected by the effect of low quantile drive and high quantile constraint. From the quantile response type, high quantile constraints are mainly distributed in the eastern coastal region, and low quantile drivers are mainly distributed in the northwest, southwest, and northeast regions, and these two driver/constraint types affect a wide range of provinces; only a few provinces are affected by the effects of low quantile drivers and high quantile constraints.

## Discussion and conclusions

This study examined tourism environmental efficiency patterns of Chinese provinces from 2000 to 2020 by using spatial analysis methods to analyze the differential patterns and spatiotemporally dependent dynamic evolutions. We aimed to further explore the factor-driven patterns of the leaps in tourism environmental efficiency through the nesting of spatiotemporal leaps and quantile regressions. Based on our findings, the following conclusions were drawn:

First, the mean value of tourism environmental efficiency in China increased and then fluctuated during the study period, showing overall spatial non-equilibrium. The results of the kernel density estimation indicate that the overall difference in tourism environmental efficiency is narrowing; however, a few high carbon emission regions show a polarization effect and the gap with other regions is increasing. In addition, the spatial autocorrelation Moran's *I* indices are all positive, indicating that inter-provincial tourism environmental efficiency has a positive spatial correlation and presents a spatial clustering phenomenon in spatial distribution.

Second, the analysis of the spatiotemporal dynamic process based on the ESTDA shows that the overall spatial association pattern of tourism environmental efficiency in China during the study period shows transfer inertia with strong path dependence or spatial locking characteristics. The eastern coastal region has a relatively stable local spatial structure and spatial dependence, whereas the central and western regions show the opposite. In addition, there are uneven characteristics between neighboring provinces in the process of spatial and temporal changes in tourism environmental efficiency, such as Tianjin, Hebei, Yunnan, Guizhou, Qinghai and Tibet. There is a certain degree of spatial and temporal competition and thus the development of low-carbon actions in tourism must give full play to the synergistic effects between regions and avoid the closure of interprovincial tourism environmental policies.

Third, the factor-driven/constrained patterns of the spatiotemporal leap in tourism environmental efficiency can be divided into four categories: openness-transportation driven, environment-resource constrained, technology-human driven, and economic-industry-urbanization constrained. The eastern coastal provinces are mainly influenced by the population-urbanization constraint model, whereas most regions in the northwest, southwest, and northeast tend to be influenced by a technology-regulation-driven model. From the northwest to the southeast, the leap in the efficiency of China's tourism environment gradually shows a stepwise pattern of "congruent constraint—reverse development—congruent development."

Fourth, environmental regulations also have a positive driving effect on carbon emissions from the tourism industry, but have not passed the significance test. The reason may be that, firstly, tourism environmental regulations, as a restraining force, will increase the pollution control costs of tourism enterprises, which may further squeeze out the R&D investment of tourism enterprises and hinder energy-saving technology innovation; Secondly, the energy consumption of the tourism industry in high-ranking areas has significantly increased during the research period and is still showing a growth trend, resulting in insufficient reflection of the regulatory effect of the tourism environment; Thirdly, it may be due to the lack of effective supervision and performance control by the government in the process of tourism environmental governance, leading to the lack of effective constraints in relevant regulatory measures.

Due to the disparity in economic development among provinces and regions, there is an obvious spatial heterogeneity in China's tourism environmental efficiency. Therefore, it is especially crucial to carry out low-carbon actions for provinces with different tourism environmental efficiency levels according to local conditions. Through an in-depth study of the spatial and temporal interaction characteristics and evolution of different patterns of tourism environmental efficiency in China, this study can better clarify the developmental stages and dynamic evolution of tourism environmental efficiency. This study also combines the deep nesting of quantile regression and spatiotemporal leaps to further clarify the differences in the implementation effects of emission reduction measures at different levels of tourism environmental efficiency, effectively avoiding the idealistic treatment mode of mean regression, better reflecting the real situation, and providing a reference for the formulation of "common but differentiated" tourism carbon reduction policies. It also provides a reference for the formulation of common, but differentiated policies for carbon reduction in tourism.

The policy insights from this study are as follows. For low-quantile constraint type areas such as Beijing, Tianjin and Hebei, which have strong spatial stability, the focus should be on the improvement and strengthening of the inhibiting factors of tourism environmental efficiency (such as population density and urbanization level). Specifically, reasonable population policies should be formulated based on regional differences, giving full play to the scale and agglomeration effects brought about by the optimization of population density, focusing on the construction of new urban areas, realizing the intensive use of public resources and service facilities, and further improving the allocation efficiency of energy resources to maintain the stability of low-carbon agglomeration. For Hainan, Shaanxi, Shanxi, and other high-quantile driven areas, it is necessary to focus on controlling and optimizing the positive drivers of tourism environmental efficiency. The service industry in these regions should focus on promoting the conversion and upgrading of traditional energy utilization technologies, vigorously developing low-carbon technologies with "green bias,” and accelerating the application and promotion of relevant low-carbon technologies to break the time lag hindrance in technology application. For high-quantile constraint type areas such as Jiangxi, Henan, Shandong, Guangdong, Guangxi, based on the nonlinear relationship between economic development and tourism environmental efficiency, we can accelerate the level of the service economy to cross the inflection point and decouple economic development from carbon emissions through the two-wheel drive of the government and market. For Hunan and Guizhou, and other low-quantile driven areas, in addition to implementing the above low-carbon technical measures, we should continue to deepen the structural reform on the supply side of the tourism industry, take the "new development concept" and "high-quality development" as the guide, prioritize the support of low-energy, low-pollution emerging We should also continue to deepen the structural reform on the supply side of the tourism industry and give priority to supporting new tourism industries with low energy consumption and low pollution, and accelerate the adjustment and optimization of the internal structure of the tourism industry. In addition, each region should actively help the tourism industry change from "double control" of energy consumption to "double control" of carbon emissions, gradually establish a mechanism for the decomposition of tourism targets, and at the same time, adjust the intensity of environmental regulations according to local conditions, and increase the policy of energy-saving and technological innovation for service enterprises. Simultaneously, the intensity of environmental regulations should be adjusted according to local conditions, and policy support for energy-saving and technological innovation in service enterprises should be increased to further reduce the cost burden of environmental regulations. In general, the low-carbon development of tourism in each region should not only consider all kinds of drivers/constraints but also combine different types of drivers/constraints and transition paths, emphasizing differentiated measures to reduce emissions in tourism and avoiding the closure of inter-provincial emission reduction policies by collaborative emission reduction between regions. At the same time, the policy implications contained in this study include: firstly, establishing a regional tourism linkage mechanism to promote the flow of tourism talents, funds, and technology, forming a pattern of regional coordination, industrial integration and complementarity, and resource co construction and sharing, further narrowing regional differences in the tourism industry, and promoting the rational agglomeration of the tourism industry in space and balanced development between regions. Secondly, by optimizing the industrial structure, we should further improve the level of tourism agglomeration and fully leverage the emission reduction effect of industrial agglomeration; Introduce energy utilization technologies and learn advanced experience in energy conservation and emission reduction in high concentration low emission zones, focus on improving energy efficiency, and continuously reduce the carbon emission intensity of the tourism industry. Thirdly, creating a favorable external environment for industrial energy conservation and emission reduction by reasonably guiding the agglomeration of regional tourism industry; we should also transform the extensive growth mode of the tourism economy, increase environmental regulations and opening-up efforts, in order to promote the intensive and low-carbon development of the tourism industry.

Due to data limitations, this study only examines changes in tourism environmental efficiency in China from 2000 to 2020. Further enrichment of sample data is needed to explore the spatial and temporal dynamic process of tourism environmental efficiency and its mechanism of action in more depth. In addition, a geographic probe model can be introduced to examine and analyze the interaction effects between different factors affecting tourism environmental efficiency to enrich and improve the influence mechanism of tourism environmental efficiency. Future research needs to integrate many factors of tourism environmental efficiency and construct a more comprehensive impact system. It can be attempted to conduct in-depth research using specific tourism destinations as classic cases to reveal their driving mechanisms from a more microscopic perspective. In terms of refinement of research content, it is necessary to further explore the dynamic correlation and spatial spillover effects of tourism environmental efficiency at different spatial scales in the future.

## Data Availability

The datasets generated during and/or analysed during the current study are available from the corresponding author upon reasonable request.
